# Cytotoxicity of Donor Natural Killer Cells to Allo-Reactive T Cells Are Related With Acute Graft-vs.-Host-Disease Following Allogeneic Stem Cell Transplantation

**DOI:** 10.3389/fimmu.2020.01534

**Published:** 2020-07-31

**Authors:** Lixia Sheng, Qitian Mu, Xiaoqing Wu, Shujun Yang, Huiling Zhu, Jiaping Wang, Yanli Lai, Hao Wu, Ye Sun, Yongxian Hu, Huarui Fu, Yi Wang, Kaihong Xu, Yongcheng Sun, Yanli Zhang, Ping Zhang, Miao Zhou, Binbin Lai, Zhijuan Xu, Minjie Gao, Yi Zhang, Guifang Ouyang

**Affiliations:** ^1^Department of Hematology, Ningbo First Hospital, Ningbo, China; ^2^Bone Marrow Transplantation Center, The First Affiliated Hospital, Zhejiang University School of Medicine, Hangzhou, China

**Keywords:** natural killer cells, cytotoxicity, CD107a, graft vs. host disease, allogeneic hematopoietic stem cell transplantation

## Abstract

**Objectives:** The mechanism and immunoregulatory role of human natural killer (NK) cells in acute graft-vs.-host-disease (aGVHD) remains unclear. This study quantitatively analyzed the cytotoxicity of donor NK cells toward allo-reactive T cells, and investigated their relationship with acute GVHD (aGVHD).

**Methods:** We evaluated NK dose, subgroup, and receptor expression in allografts from 98 patients who underwent allogeneic hematopoietic stem cell transplantation (allo-HSCT). A CD107a degranulating assay was used as a quantitative detection method for the cytotoxic function of donor NK cells to allo-reactive T cells. In antibody-blocking assay, NK cells were pre-treated with anti-DNAM-1(CD226), anti-NKG2D, anti-NKP46, or anti-NKG-2A monoclonal antibodies (mAbs) before the degranulating assay.

**Results:** NK cells in allografts effectively inhibited auto-T cell proliferation following alloantigen stimulation, selectively killing alloantigen activated T cells. NKG2A^−^ NK cell subgroups showed higher levels of CD107a degranulation toward activated T cells, when compared with NKG2A^−^ subgroups. Blocking NKG2D or CD226 (DNAM-1) led to significant reductions in degranulation, whereas NKG2A block resulted in increased NK degranulation. Donor NK cells in the aGVHD group expressed lower levels of NKG2D and CD226, higher levels of NKG2A, and showed higher CD107a degranulation levels when compared with NK cells in the non-aGVHD group. Using univariate analysis, higher NK degranulation activities in allografts (CD107a^high^) were correlated with a decreased risk in grade I–IV aGVHD (hazard risk [HR] = 0.294; *P* < 0.0001), grade III–IV aGVHD (HR = 0.102; *P* < 0.0001), and relapse (HR = 0.157; *P* = 0.015), and improved overall survival (HR = 0.355; *P* = 0.028) after allo-HSCT. Multivariate analyses showed that higher NK degranulation activities (CD107a^high^) in allografts were independent risk factors for grades, I–IV aGVHD (HR = 0.357; *P* = 0.002), and grades III–IV aGVHD (HR = 0.13; *P* = 0.009).

**Conclusions:** These findings reveal that the degranulation activity of NK in allografts toward allo-activated T cells was associated with the occurrence and the severity of aGVHD, after allogeneic stem cell transplantation. This suggested that cytotoxicity of donor NK cells to allo-reactive T cells have important roles in aGVHD regulation.

## Introduction

Natural killer (NK) cells are the first donor-derived subset of lymphocytes that are reconstructed following allogeneic hematopoietic stem cell transplantation (allo-HSCT). Although the roles of NK cells in preventing relapse and infection after allo-HSCT for hematologic malignancies has been well established (1–4), the function of human NK cells in acute graft-vs.-host-disease (aGVHD), which is a common complication of allo-HSCT, is still equivocal.

Some studies have demonstrated that killer immunoglobulin-like receptor (KIR)-ligand mismatches trigger donor vs. recipient NK cell allo-reactivity, suppressing the development of aGVHD by ablating host antigen-presenting cells (APCs), which are essential for the activation of donor T cell in aGVHD (5–7). However, many studies have failed to prove the beneficial effect of allo-reactive NK cells on aGVHD (8–12). Similarly, conflicting results from clinical studies also hint at other mechanisms for the regulation of aGVHD by NK cells (13).

The function that NK cells can distinguish target cells from healthy cells is controlled by integrating signals from inhibitory and activating receptors (14–18). Donor NK allo-reactivity, which is based on the lack of ligands for donor KIR in the recipient, can lead to NK cell activation though “missing-self” recognition (19–21). When target cells are exposed to stress, such as viral infection, the ligands for activating NK cell receptors are upregulated, binding to NK activating receptors and activate NK cells via “induced-self” recognition (22–24). Studies have demonstrated that activated T cells up-regulate the expression of ligands for activating NK cell receptors, making them vulnerable to NK cell killing though the “induced-self” model (25, 26). As donor NK and T cells share similar trafficking routes after allo-HSCT (27), and recent studies have shown that NK cells exert cytotoxicity toward activated T cells (28, 29), the NK cell–mediated direct lysis of allo-reactive T cells through the “induced-self” model may present an important mechanism for aGVHD regulation by NK cells. Olson et al. proved this hypothesis in a major histocompatibility complex (MHC)-mismatched mouse bone marrow transplantation (BMT) model (30). However, we know little about the role of NK cell cytotoxicity toward allo-reactive T cells in human aGVHD.

In this study, we investigated the role of NK cells in the regulation of T cell allo-reactivity in human allo-HSCT, and demonstrated that cytotoxicity of donor NK cells toward allo-reactive T cells was associated with the occurrence of overall and grade III–IV aGVHD.

## Materials and Methods

### Patients and Samples

Ninety-eight consecutive patients with acute lymphoid leukemia (ALL), acute myeloid leukemia (AML), myelodysplastic syndrome (MDS), non-Hodgkin's lymphoma (NHL), or chronic myeloid leukemia (CML) underwent allo-HSCT and were included in this study. Among these, 37 patients underwent human leukocyte antigen (HLA)-matched related HSCT, 13 patients underwent HLA-matched unrelated HSCT, and 48 patients underwent HLA-haplo-identical related HSCT. Stem cell sources were peripheral blood stem cells without T-cell depletion. The prophylaxis regimens for GVHD were cyclosporine A, short-term methotrexate, and mycophenolate mofetil. In addition, ATG was added to HLA-matched unrelated and HLA-haplo-identical related HSCT. The high risk disease status at the time of HSCT was defined as > second remission, or acute leukemia without remission after two cycles of induction chemotherapy, refractory anemia with excess blasts, and blast crisis of chronic myelomonocytic leukemia. KIR-ligand mismatch was evaluated based on donor and recipient HLA gene typing. The characteristics of the 98 patients and corresponding donors are summarized in Table 1. All samples in this study were collected from donor granulocyte-colony stimulating factor (G-CSF) mobilized peripheral blood stem cell (PBSC) harvests before transplantation. All patients and donors provided written informed consent. The study was approved by the Clinical Ethics Review Committee at Ningbo First Hospital and was performed in accordance with the Declaration of Helsinki.

**Table 1:** Patient, donor, disease, and transplantation characteristics.

	**Non-aGVHD**	**aGVHD**	***P* value**
*N*	47	51	–
Patient age	38 (15-63)	40 (14-65)	0.177
Patent sex (M:F)	25:22	26:25	0.827
Diagnosis			0.156
ALL	5	14	
AML	16	14	
MDS	15	17	
NHL	8	3	
CML	3	3	
High risk, no. (%)	12	21	0.102
Donor source			0.051
MRD	22	15	
Haplo-identical	17	31	
MUD	8	5	
Donor/patient sex			0.199
M–>M	15	8	
M–>F	12	18	
F–>M	10	12	
F–>F	10	13	
Conditioning			0.439
MA	45	51	
RIC	2	0	
GVHD prophylaxis			0.076
MTX + CSA + MMF + ATG	25	36	
MTX + CSA + MMF	22	15	
KIR-L GVH mismatch	18	21	0.77
Cell composition in allografts, median (range)			
CD34^+^ cells, ×10^6^/kg	6.1 (2.05~16.73)	5.3 (1.58~12.40)	0.197
CD3^+^ cells, ×10^8^/kg	1.88 (0.43~4.07)	1.78 (0.35~4.78)	0.347
CD56+ cells, ×10^7^/kg	3.38 (0.29~6.45)	2.68 (0.27~7.10)	0.059
NK:T ratio	0.225 (0.051~0.498)	0.172 (0.049~0.698)	0.117

*GVHD, graft-vs.-host disease; F, female; M, male; AML, acute myeloid leukemia; ALL, acute lymphoblastic leukemia; MDS, myelodysplastic syndrome; NHL, non-Hodgkin's lymphoma; CML, Chronic Myelogenous Leukemia; MRD, matched related donor; MUD, matched unrelated donor; KIR-L, killer Ig-like receptor ligand; NK, natural killer cell; T, T cell*.

### mAbs and Flow Cytometry Analyses

NK cells were characterized by FITC-conjugated anti-human CD56, PE-conjugated anti-human CD16, and APC-conjugated anti-human CD3 mAbs (Becton Dickinson, San Diego, CA, USA). To analyze the expression of receptors on NK cells, the following mAbs were used: APC-conjugated anti-NKG2D (BAT221 clone), PE-conjugated anti-human NKp46 (BAB281 clone), and FITC-conjugated anti- human DNAM-1 (F22 clone) (all Becton Dickinson). PE conjugated anti-human NKG2A was purchased from Beckman Coulter (Fullerton, CA, USA). A Beckman Coulter flow cytometer, FC5000 (Fullerton, CA, USA), was used to analyze samples.

### CD56^+^ NK Cell and CD3^+^T Cell Isolation and Proliferation Assays

Mononuclear cells (MNCs) were isolated from each G-CSF mobilized PBSC harvest by Ficoll-Hypaque (MultiSciences Biotech, Hangzhou, China) density centrifugation. CD56^+^ NK cells and CD3^+^T cells were isolated from MNCs by positive selection, using FACS (Fluorescence activated cell sorting), and used for the following experiments.

For proliferation assays, carboxyfluorescein diacetate succinimidyl ester (CFSE, Invitrogen, Carlsbad, CA, USA) -labeled CD3^+^T cells (2 × 10^5^ cells/well) were stimulated with phytohemagglutinin (PHA), anti-CD3/anti-CD28, or allogeneic dendritic cells (allo-DCs) separately in 200 μl RPMI 1640 medium containing 10% fetal bovine serum (FBS), in a 96-well micro-plate (day 0). NK cells from the same donor were added to the culture at different NK/T ratios (0:10 to 1:5). At day four, cells were stained with a PECY7-conjugated anti-CD3 mAb (Becton Dickinson), and the proliferation of CD3^+^ T cells was analyzed by detecting diluted CFSE signals with flow cytometry.

### Functional Assessments of NK Cells

For degranulation assays, NK cells and anti-CD3/anti-CD28 mAbs activated T cells from the same donor were co-cultured at an NK to T cell ratio of 1:1, for 4 h at 37°C, in the presence of APC-conjugated anti-human CD107a [lysosomal-associated membrane protein (LAMP)-1] mAb (H4A3, BD Biosciences, San Jose, CA) and GolgiStop^TM^ containing monensin (BD Biosciences). In blocking assays, NK cells were incubated with blocking antibodies for 20 min before being co-cultured with target cells. The following anti-human mAbs were added at 10 μg/mL: NKG2D (clone 149810; R&D Systems, Minneapolis, MN, USA), NKG2A (clone NNC0141-0100, R&D Systems), DNAM-1 (clone DX11, BioLegend, San Diego, CA, USA), and NKP46 (BioLegend). Mouse IgG1 mAbs (R&D Systems) served as isotype-matched control mAbs. The expression of CD107a in NK cells was measured by flow cytometry.

For intracellular cytokine staining, NK cells were co-cultured with the unstimulated T cells or activated T cells for 4 h, and GolgiStop™ was added to trap protein in the cytoplasm. Monoclonal antibodies APC-conjugated anti-human CD56 mAb, FITC-conjugated anti-human IFN-γ, PE-conjugated anti-human TNF-α, FITC-conjugated anti-human TGF-β, and PE-conjugated anti-human IL-10 (BD Bioscience) were used for cell surface marker and in-tracellular cytokine staining. The intracellular cytokine level of NK cells was detected by flow cytometry. The granzyme B were quantified by ELISA in supernatants after co-culture of NK cells with the unstimulated T cells or activated T cells for 4 h.

For *in vitro* cytotoxicity assays, a CFSE-7AAD (7-Aminoactinomycin D, BD Pharmingen, San Diego, CA, USA) based flow cytometric cytotoxicity assay was performed using CFSE-labeled T cells stimulated for 4 d with allo-DCs as targets, and autogeneic NK cells as effectors. In brief, effector and target cells were co-cultured at E:T ratios of 50:1, 25:1, 10:1, 5:1, for 4 h at 37°C. Cells were then washed and labeled with PECY7 conjugated anti-CD3 mAb, and 7AAD (5 μg/mL) for 20 min and analyzed by flow cytometry.

### Statistical Analysis

Patient characteristics in aGVHD and non-aGVHD groups were compared by the χ2-test for categorical variables or the Mann–Whitney U-test for continuous variables. Student's *t*-tests or a two-way ANOVA analyses were used to compare receptor expression, and degranulation activities of NK cells among groups. The optimal cut-off point of CD107a expression in donor NK cells was identified using the receiver operating characteristic (ROC) curve. Overall survival (OS) was estimated by the Kaplan–Meier method. The Gray's test was applied for comparisons of cumulative incidences of acute GVHD and relapse. Death, without aGVHD, was defined as the competing event for aGVHD, while relapse-free mortality was the competing event for relapse. The Cox regression model was employed for univariate and multivariate analyses. Risk factors for univariate analysis included the age of recipient and donor, the gender of recipient and donor, diagnosis, KIR-L mismatch/match between donor and recipients, donor source, high risk disease before transplantation, the dose of CD56^+^ NK cells, CD34^+^cells, and CD3^+^T cells, the NK/T cell ratio; the CD56^dim^/CD56^bright^ ratio, NKG2A^+^ proportion, levels of CD226, NKG2D and NKP46 expression of NK cells, and NK CD107a degranulation activity in allografts. All covariates with *P* < 0.10 during univariate analysis were further included in a multivariate Cox regression model. All tests were bilateral, and a difference was considered significant when *P* < 0.05. Statistical analyses were performed on SPSS 25 statistical software (IBM, Armonk, NY, USA), and R 3.6.2 statistical software (https://www.r-project.org/) was employed to calculate the cumulative incidences, when considering the presence of competing risks. All calculated averages were defined as the parametric mean ± SD. ^**^P < 0.01.

## Results

### Patient Characteristics

Ninety-eight donor PBSC samples from 98 patients receiving allo-HSCT were analyzed in this study. Patient characteristics are shown in Table 1. No significant differences were observed in patient age, patient sex, gender matching between donors and recipients, underlying disease, donor source, conditioning regimen, serotherapy, KIR-L mismatch, and dose of CD34^+^, CD3^+^, or CD56^+^ cells in allografts between the GVHD group and the non-aGVHD group. The median duration follow-up was 412 d (range; 71–1,320 d) after transplantation. All 98 patients achieved engraftment and complete donor chimerism after transplantation. The chimerism dynamics of donor NK and T cells were shown (Figure S1). Grades I, II, III, and IV aGVHD occurred in 16, 16, 14, and 5 cases, respectively. Of 24 patients that died, nine died from severe infection, two died from severe gastrointestinal aGVHD with pulmonary infection, and 13 relapsed.

### NK Cells in Allografts Inhibited T Cell Proliferation and Exhibited Cytotoxicity Against Allo-Reactive T Cells

**Figure 1:**
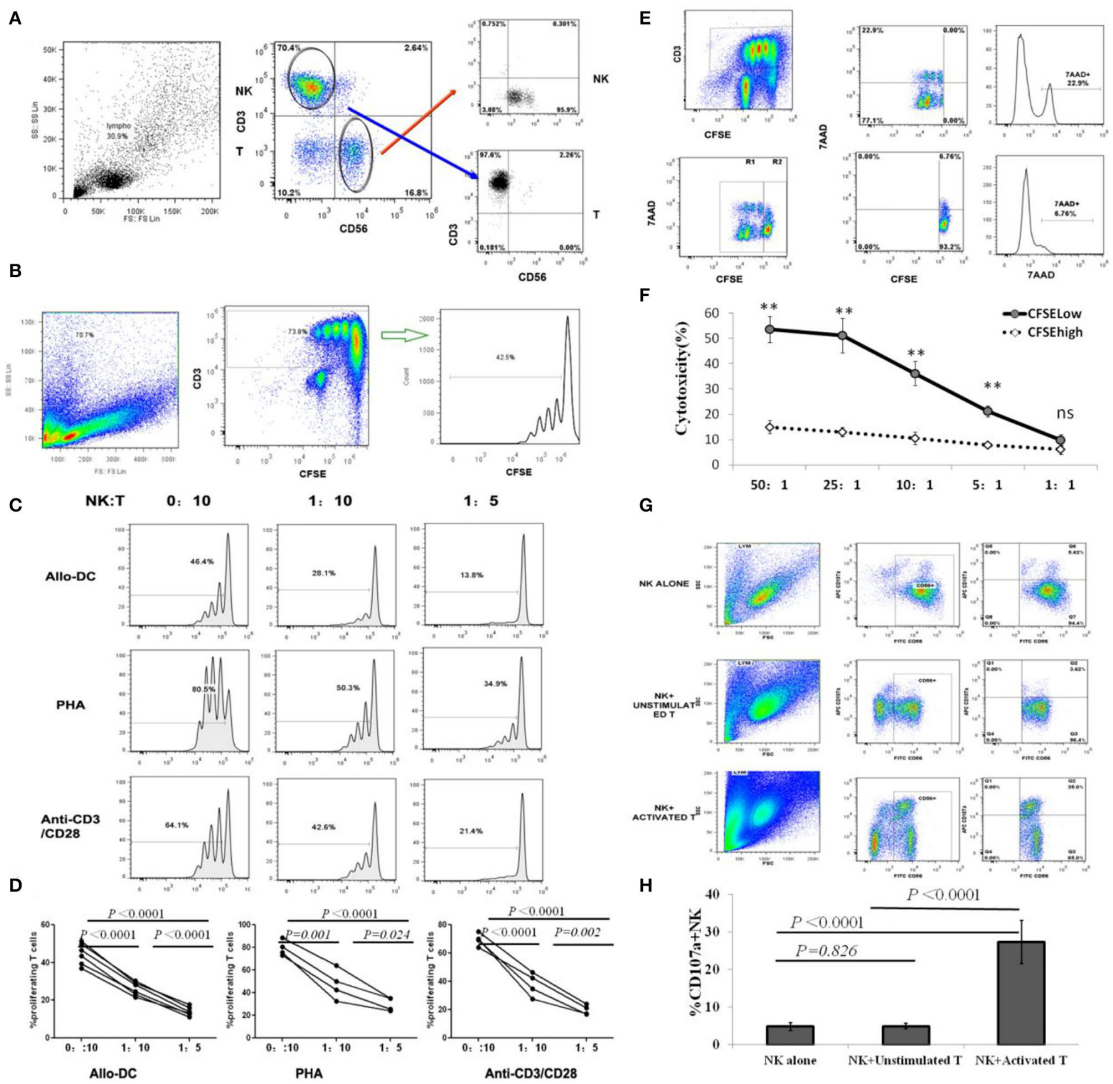
NK cells inhibit T cell proliferation by selectively killing alloantigen activated T cells. **(A)** Representative gating strategy for NK and T cell sorting; **(B)** Representative gating strategy for T cell proliferation assay. **(C,D)** CFSE-labeled CD3^+^T cells were stimulated with PHA, anti-CD3/anti-CD28 mAbs or allo-DCs, and autologous CD56^+^ NK cells were added at NK/T ratios of 0:10, 1:10, or 1:5. Four days later, CD3^+^T cell proliferation was analyzed by flow cytometry. The percentage of proliferating T cells was defined by CFSE intensities (*n* = 4). **(E,F)** CFSE-labeled CD3^+^T cells were first stimulated with allo-DCs for 96 h and then used as target cells for NK killing assays at effector:target (E:T) ratios of 50:1, 25:1, 10:1, 5:1, or 1:1. Allo-reactive T cells were distinguished by lower CFSE intensity (CFSE^low^) in CD3^+^T cells. 7AAD was labeled to identify dead cell and analyzed by flow cytometry (*n* = 4). **(G,H)** Naïve T cells or T cells activated by anti-CD3/anti-CD28 mAbs were co-cultured with NK cells at an effector:target (E:T) ratio of 1:1 for CD107a degranulating assay. NK cells cultured alone were used as controls. The percentage of CD107a^+^ in CD56^+^NK cells represented the level of NK degranulation toward T cells (*n* = 4). All calculated averages were defined as the parametric mean ± SD. Student's *t*-tests, or two-way ANOVA analyses, were used to compare the mean among groups. ns: not significant. **P < 0.01.

Olson et al. demonstrated that donor NK cells could inhibit and kill alloantigen activated T cells during the development of acute GVHD in their mouse model, indicating that donor NK cell mediated inhibition and lysing of activated donor T cells may represent an important mechanism for NK cell–mediated aGVHD reduction (30). However, the direct modulation of donor allo-reactive T cell responses by autogeneic NK cells in human GVHD has not been fully investigated. For donor T-cell proliferation, activation is the core immunopathophysiology of aGVHD; therefore, we investigated the effects of donor NK cells on the proliferation of autogeneic CD3^+^T cells, following activation by PHA, anti-CD3/anti-CD28 mAbs, or allo-DCs derived from recipients. CFSE-labeled resting CD3^+^T cells were stimulated by PHA, anti-CD3/anti-CD28 mAbs, or allo-DCs (T/DC = 5:1), and co-cultured with autologous CD56^+^ NK cells at NK/T ratios of 0:10, 1:10, or 1:5. Ninety-six hours later, the percentage of proliferating CD3^+^T cells was detected by flow cytometry (Figures 1A,B). As shown in Figures 1C,D, the proliferation of T cells, as defined by CFSE dilution, was significantly inhibited by donor NK cells, in a NK cell dose dependent pattern.

To further validate that NK cell-mediated cytotoxicity against T cells led to the suppression of alloantigen-activated T cell proliferation by autologous NK cells, CFSE-labeled resting CD3^+^T cells were stimulated with allogeneic dendritic cells (allo-DCs) for 96 h, and then used as target cells for an NK killing assay. Our results revealed allo-reactive T cells were distinguished by lower CFSE intensity (CFSE^low^) in CD3^+^T cells (Figure 1E). Flow cytometric analysis using 7AAD to identify dead cells revealed that donor NK cells mainly killed proliferating T cells (CFSE^low^), but not non-proliferating T cells (CFSE^high^), in a cell dose-dependent manner at effector:target (E:T) ratios of 50:1, 25:1, 10:1, or 5:1 (Figures 1E,F).

In the process of NK degranulation, lysosomal associated membrane protein-1 (LAMP-1, CD107a) on the surface of lysosomal granules is transported to the cell surface and can be used for antibody binding studies. This allows for the recognition of activated NK cells, making them attractive biomarkers for assessing granulocytic exocytosis and cytotoxic activity of NK cells (26, 27). As shown (Figures 1G,H), donor NK cells displayed degranulation activity to activated but not resting T cells, which was consistent with NK cells killing activated proliferating T cells instead of resting T cells, in the killing assay. In addition to CD107a degranulation, the Granzyme B concentration in NK and activated T cell co-cultures was significantly higher (1422.25 ± 256.77 pg/ml) than that in NK and unstimulated T cell co-cultures (782.75 ± 161.77 pg/ml) (*P* = 0.014). However, there was no difference in cytokines IFN-γ, TNF-α, IL-10, and TGF-β secreted by NK cells after co cultured with activated or unstimulated T cells (Figure S2). Therefore, NK cells selectively killed activated T cells and played an inhibitory role on T cell proliferation induced by alloantigen stimulation.

### The Effects of NKG2A^+^/NKG2A^−^ Subsets and Receptor Expression on NK Cell Cytotoxicity Against T Cells Are Associated With aGVHD After Allo-HSCT

As the CD107a degranulation assay is more feasible than the killing assay, we performed a CD107a degranulation as**s**ay to identify the cytotoxic effects of NK cells to activated T cells, for all PBSC donors.

**Figure 2:**
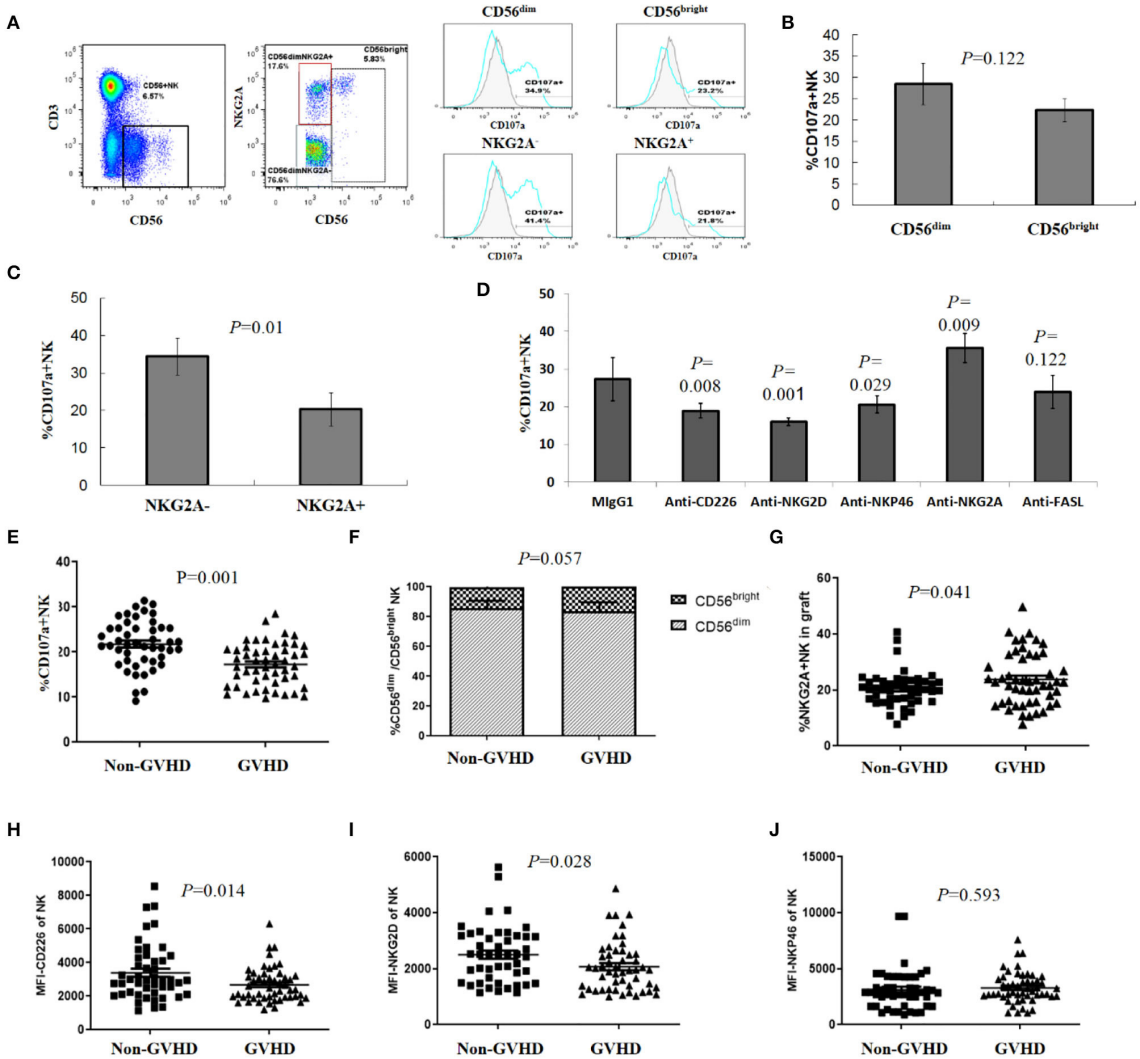
Subgroup and receptor expression of donor NK cells affected NK degranulation toward activated T cells associated with aGVHD. **(A)** Representative gating strategy. CD56^dim^ and CD56^bright^ NK cells were gated and subsets were defined based on the expression of NKG2A, the percentage of CD107a positive cells was analyzed on each subset of NK cells. **(B)** CD107a expression in CD56^dim^ and CD56^bright^ NK cells (*n* = 4), **(C)** CD107a expression in NKG2A^−^ and NKG2A^+^ subgroups (*n* = 4), **(D)** NK cells were pretreated with neutralizing antibodies (or relevant isotype-matched Ig controls) before degranulation assay (*n* = 4). **(E)** Levels of donor NK degranulation toward activated T cells were significantly lower in the aGVHD group than in the non-aGVHD group (*P* = 0.001, *n* = 98). Percentage of CD56^dim^ and CD56^bright^ NK cells **(F)**, NKG2A^+^ NK cells **(G)** in allografts from the aGVHD and non-aGVHD groups (*n* = 98). MFI of CD226 **(H)**; NKG2D **(I)** and NKP46 **(J)** of NK cells in allografts from aGVHD and non-aGVHD groups (*n* = 98). All calculated averages were defined as the parametric mean ± SD. Student's *t*-tests or two-way ANOVA analyses were used to compare the mean among groups.

We further investigated differences in NK degranulation against autologous activated T cells between CD56^dim^ and CD56^bright^, and NKG2A^+^ and NKG2A^−^ subsets. As shown (Figures 2A–C), the degranulation of CD56^dim^ NK cells toward autologous activated T cells was stronger than the CD56^bright^ subset, and NKG2A^−^ NK cells were degranulated more potently than the NKG2A^+^ subgroup, suggesting that subgroup distribution patterns of donor NK cells influenced NK cytotoxicity against activated T cells.

The cytotoxicity of NK cells is regulated by signal integration from a complex repertoire of activating and inhibiting receptors (14, 17, 18). According to the NK education and tolerance hypothesis (31–33), it is impossible for NK cells to kill auto-T cells by KIR-L mismatching. Therefore, we further analyzed the potential roles of NK activating receptors by blocking interactions between NK activating receptors and corresponding ligands with neutralizing antibodies, before the degranulation assay. We observed that blocking NKG2D, DNAM-1 (CD226), or NKP46 led to significant decreased degranulation (CD107a expression) of NK cells toward activated auto-T cells. Accordingly, we also found that the expression of NKG2D ligands (MICA/MICB, ULBP-1, ULBP-3) and DNAM-1 ligands (PVR) on T cell surface was up-regulated after activation (Figure S3). On the contrary, blocking the HLA-E–NKG2A interaction with an anti-NKG2A mAb resulted in increased degranulation (Figure 2D). These results suggested that activated receptors NKG2D, DNAM-1 (CD226), and NKP46 played important roles in triggering NK cell cytoxicity, while NKG2A, an inhibitory receptor of NK cells, played a negative role in NK cell cytotoxicity toward allo-reactive auto-T cells.

NK cells may kill target cells by means other than perforin-mediated cytotoxicity. As T cells could upregulated expression of Fas/FasL after activation and Fas/FasL pathway has been proved to participate NK cell-mediated cytotoxicity against tumor cells (34, 35), we addressed whether FAS/FAS-L pathway was implicated in NK cell killing of allo-reactive T cells. However, blocking FasL did not affect the degranulation and killing of NK cells to allo-antigen activated T cells (Figure 2D). NK degranulation varied between donors, with an average 17.26 ± 4.69% donor NK cells of the aGVHD group showing degranulation activity toward autologous activated T cells, when compared to 21.78 ± 5.26% NK cells in the non–aGVHD group (*P* = 0.001) (Figure 2E).

Furthermore, we evaluated NKG2A^+^ and NKG2A^−^, CD56^dim^ and CD56^bright^ subsets and receptor expression on CD56^+^ NK cells in patient allografts in aGVHD and non-aGVHD groups. When analyzing NKG2A expression, we observed that 23.8 ± 9.47% donor NK cells for aGVHD patients were positive for NKG2A, when compared with 20.42 ± 6.2% NK cells in the non-aGVHD group (Figure 2G, *P* = 0.041). The differences in CD56^dim^ and CD56^bright^ subset proportions between groups were not statistically significant (Figure 2F). After this, we analyzed the differences in NK activating receptors, CD226, NKG2D, and NKP46, which have been shown to enhance NK killing activity to activated T cells *in vitro* (29, 36), in allografts between aGVHD and non-aGVHD groups. We observed that the expression of DNAM-1 (CD226) and NKG2D in donor NK cells of the aGVHD group was higher than that of the non aGVHD group, while differences in NKP46 expression between the groups were not statistically significant (Figures 2H–J).

### CD107a Expression (>20.5%) in Donor NK Cells Is an Independent Predictor of aGVHD

**Figure 3:**
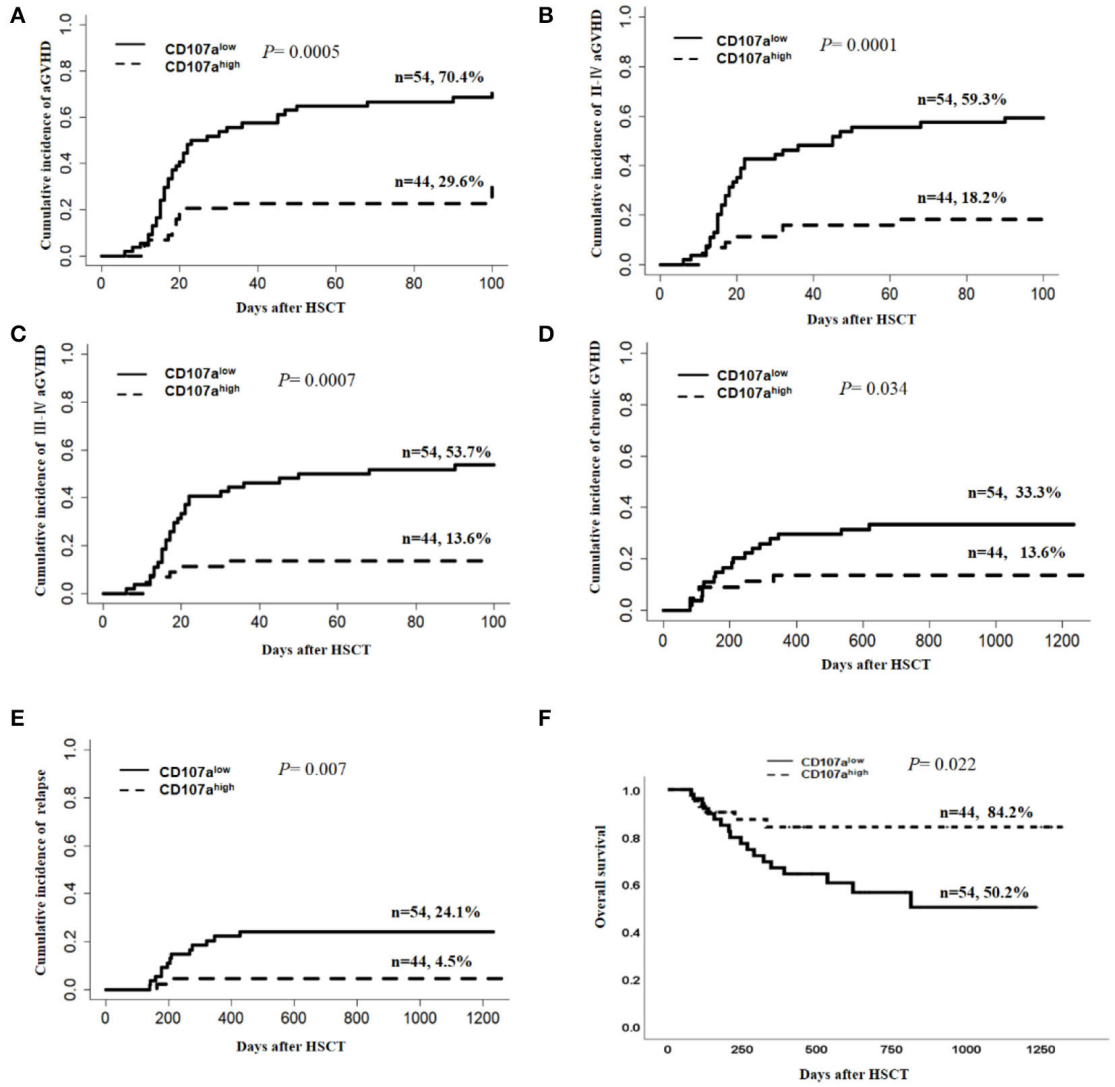
Donor NK CD107a degranulation toward activated T cells was predictive for risk of aGVHD, chronic GVHD, relapse, and overall survival. The Gray's test was applied for comparisons of cumulative incidences of acute GVHD, chronic GVHD, and relapse. Death, without aGVHD, was defined as the competing event for aGVHD, while relapse-free mortality was the competing event for relapse. Cumulative incidence estimates of grade I–IV aGVHD **(A)**, gradeII–IV aGVHD **(B)**, grade III–IV aGVHD **(C)**, chronic GVHD **(D)**, and relapse **(E)** or Kaplan–Meier survival estimates for overall survival **(F)** for patients in “CD107a^low^” and “CD107a^high^” groups, separated according to the optimal cutoff of 20.5% for donor NK CD107a degranulation toward activated T cells.

Using the receiver operating characteristic (ROC) curve, we selected a cut-off of 20.5% for CD107a expression in donor NK cells in the degranulation assay, which provided a sensitivity of 75% and a specificity of 64% for the prediction of aGVHD. Based on CD107a expression in donor NK cells to activated T cells, patients were divided into the CD107a^high^ group (*n* = 54) and the CD107a^low^ group (*n* = 44). When compared with the CD107a^low^ group, patients in the CD107a^high^ group showed lower incidences of overall aGVHD (29.6 vs. 70.42%, *P* = 0.0003, Figure 3A), grade II–IV aGVHD (18.2 vs. 59.3%, *P* = 0.0001, Figure 3B) and grade III–IV aGVHD (13.6 vs. 53.7%, *P* = 0.0007, Figure 3C).

**Figure 4:**
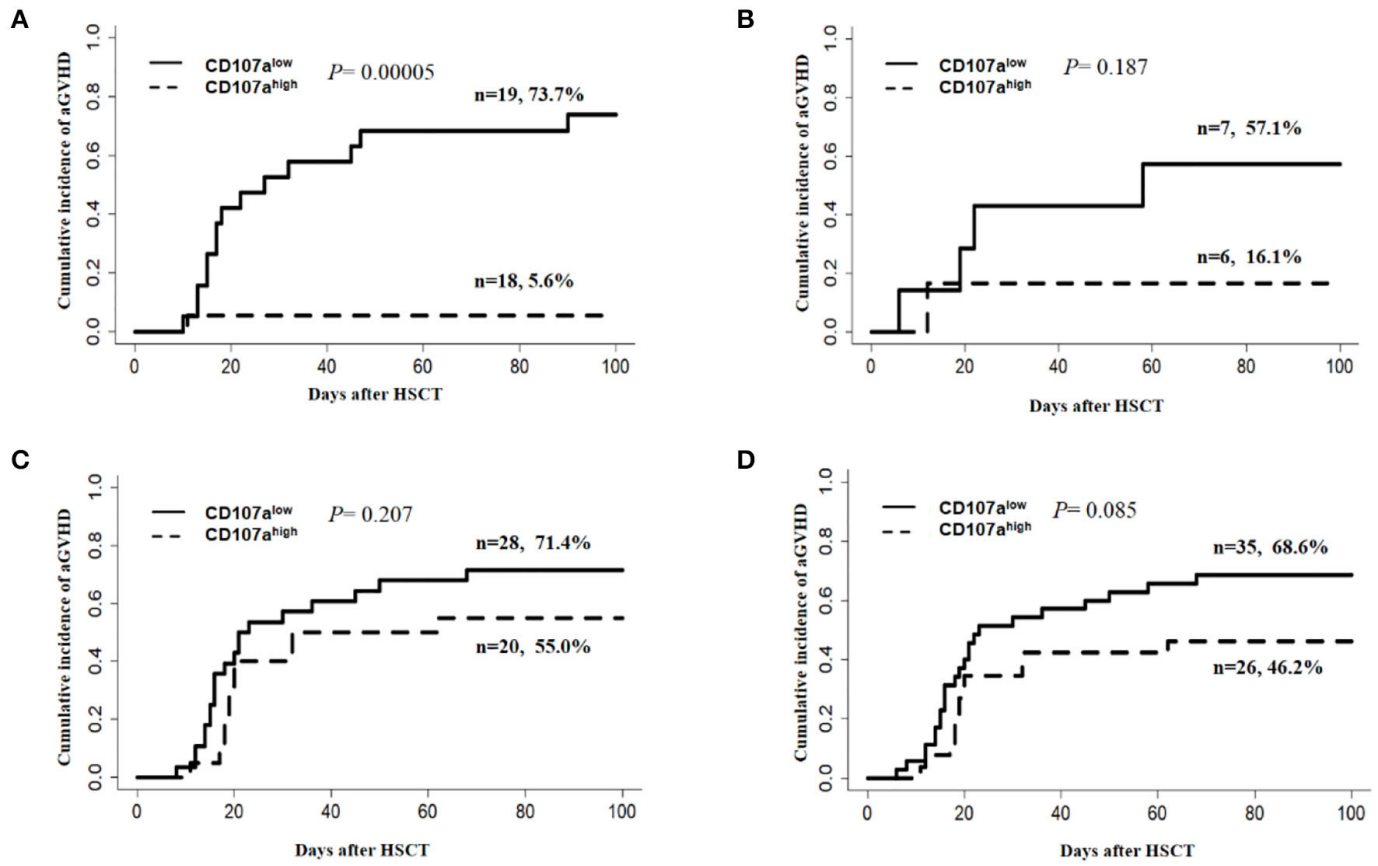
Subgroup analysis for predictive value of the donor NK CD107a degranulation toward activated T cells for grade I–IV aGVHD. **(A)** HLA-matched related HSCT(MRD), **(B)** HLA-matched unrelated HSCT(MUD), **(C)** HLA-haplo-identical related HSCT. **(D)** Patients with ATG for the prophylaxis of GVHD.

Considering the potential influence of the donor source and ATG use on the development of aGVHD, subgroup analysis was carried out. In relation to the donor source, the CD107a^high^ group demonstrated a lower cumulative incidence of overall aGVHD than the CD107a^low^ group when the donor was HLA-matched related (MRD) (5.6 vs. 73.7%; *P* = 0.0005; Figure 4A), but this effect was not seen in HLA-matched unrelated donors (MUD) (16.1 vs. 57.1%; *P* = 0.187; Figure 4B), and haplo-identical donors (55 vs. 71.4%; *P* = 0.207 Figure 4C). In 61 patients who received HLA-matched unrelated and HLA-haplo-identical related HSCT, additional ATG was used. The predictive value of CD107a expression in donor NK cells for overall aGVHD was not statistically significant when ATG was added(46.2% vs. 68.6%; *P* = 0.085 Figure 4D). Considering ATG was only used in HLA-matched unrelated and HLA-haplo-identical related HSCT in our study, we speculated that the main reasons why the predictive value of the donor NK CD107a degranulation towards activated T cells for overall aGVHD was not significant in MDR and haplo-identical HSCT might be that ATG weakened NK cell function (37) and that each subgroup had relatively small cases.

In univariate analyses, besides CD107a, other factors also predicted a reduced grade I–IV aGVHD risk, the dose of infused NK cells > 2.19 × 10^7^/kg (HR = 0.551; *P* = 0.037), and median fluorescent intensity (MFI) of NKG2D on NK cells > 2491 in allografts (HR = 0.471; *P* = 0.015) (Table 2). Other factors predicting decreased grade III–IV aGVHD included, matched related donors vs. haplo-identical donors (HR = 0.504; *P* = 0.033), and the percentage of NKG2A+NK ≤ 25.5% in allografts (HR = 0.297; *P* = 0.008). In univariate analysis, the non-statistically significant factors for predicting aGVHD included the age and gender of recipients and donors, diagnosis, high risk disease before transplantation, the KIR-L mismatch between donors and recipients, additional usage of ATG for GVHD prophylaxis, the dose of CD34^+^ cells, CD3^+^T cells, the NK/T cell ratio, the CD56^dim^/CD56^bright^ NK cell ratio, and DNAM-1 and NKP46 expression levels of NK cells in allografts.

**Table 2:** Univariate and multivariable analysis of risk factors for clinical outcomes of allogeneic stem cell transplantation.

**Covariate**	**Univariate analysis**			**Multivariate analysis**		
	**HR**	**95% CI**	***P***	**HR**	**95% CI**	***P***
**GRADE I–IV aGVHD**						
NK dose: >2.19 vs. ≤ 2.19 × 10^7^/kg	0.551	0.315–0.963	0.037			
With ATG vs. without ATG	0.603	0.33–1.101	0.096			
NK CD107a level: >20.5 vs. ≤ 20.5%	0.294	0.156–0.554	0.000	0.357	0.184–0.69	0.002
NKG2A + %NK: >25.5 vs. ≤ 25.5%	1.648	0.927–2.931	0.089			
MFI-CD226 of NK: >3,589 vs. ≤ 3,589	0.492	0.231–1.048	0.066			
MFI-NKG2D of NK: >2,491 vs. ≤ 2,491	0.471	0.257–0.862	0.015	0.384	0.285–0.721	0.003
**GRADE III–IV aGVHD**						
MRD vs. Haplo	0.504	0.268–0.946	0.033			
NK dose: >2.19 vs. ≤ 2.19 × 10^7^/kg	0.428	0.173–1.055	0.094			
NK CD107a level: >20.5 vs. ≤ 20.5%	0.102	0.024–0.445	0.002	0.13	0.029–0.595	0.009
NKG2A + %NK: >25.5 vs. ≤ 25.5%	3.368	1.372–8.355	0.008	3.627	1.466–0.026	0.005
MFI-NKG2D of NK: >2,491 vs. ≤ 2,491	0.403	0.145–1.123	0.082			
**CHRONIC GVHD**						
NK CD107a level: >20.5 vs. ≤ 20.5%	0.503	0.248–1.019	0.034			
Non-aGVHD vs. aGVHD	2.134	1.065–4.279	0.033			
**RELAPSE**						
High risk	9.185	2.905–29.035	0.000	6.924	1.922–24.941	0.003
Donor NK CD107a level	0.157	0.035–0.696	0.015			
**OS**						
High risk	4.229	1.865–9.588	0.001	3.619	1.573–8.325	0.002
0–II aGVHD vs. III–IV aGVHD	0.124	0.038–0.405	0.002	2.934	1.253–6.870	0.013
NK CD107a level: >20.5 vs. ≤ 20.5%	0.355	0.14–0.895	0.028			

Multivariate Cox regression models were applied to evaluate the prognostic value of CD107a expression in donor NK cells in allografts. All variables used for the Cox model had a univariate *p*-value < 0.1. As shown (Table 2), CD107a expression in donor NK cells > 20.5%, was an independent predictor for the grade I–IV aGVHD (HR = 0.357; *P* = 0.002), and grade III–IV aGVHD (HR = 0.13; *P* = 0.009).

In univariate analysis, the CD107a^high^ group demonstrated a lower cumulative incidence of cGVHD than the CD107alow group (13.6 vs. 33.3%; *P* = 0.034; Figure 3D). The cumulative incidence of relapse in the CD107a^high^ group was lower than the CD107a^low^ group (4.5 vs. 24.1%; *P* = 0.007; Figure 3E). There was no difference in the cumulative incidence of non-relapse mortality (NRM) between the CD107a^high^ group and the CD107a^low^ group (*P* = 0.46). The 2-year overall survival was 84.2% in the CD107a^high^ group, while that of the CD107a^low^ group was 50.2% (*P* = 0.022; Figure 3F). However, multivariate analyses showed that the predictive value of CD107a expression in donor NK cells for chronic GVHD, relapse and overall survival was not statistically significant (Table 2).

## Discussion

There is growing evidence that NK cells have immunomodulatory functions and can inhibit the immune responses of T cells (34, 35, 38–43). Donor T cell activation is the core immunopathophysiology mechanism in acute graft vs. host disease. Studies have demonstrated that donor NK cells inhibit the proliferation of T cells and show cytotoxicity to activated T cells in a mouse aGVHD model (30, 44). However, the direct regulation of donor allo-reactive T cell responses by autogeneic NK cells in human GVHD has not been fully investigated. In this study, we demonstrated that NK cells negatively regulate T cells response to allo-DCs in humans, which was consistent with a previous report in a murine model (30). NK cytotoxicity against alloantigen activated T cells may suggest an important mechanism whereby NK cells regulate T cell allo-reactivity in human aGVHD.

The observation that NK cells are capable of regulating T cell allo-reactivity, which has been validated in *in vitro* studies and animal models, should be explored in clinical transplantation models. In this study, the relationship between the killing effects of donor NK cells to activated T cells and the incidence of aGVHD was explored in a group of allogeneic hematopoietic stem cell transplantation patients. We established a method to detect the cytotoxic functions of donor NK cells toward activated T cells, through CD107a degranulation analysis. Our study demonstrated that the cytotoxic effects of donor NK cells toward activated T cells was related to the occurrence and severity of aGVHD in human HSCT. We observed that the degranulation activity of donor NK cells in the non-aGVHD group was higher when compared to donor NK cells in the aGVHD group. Furthermore, the high degranulation activity of donor NK cells significantly decreased the rate of overall aGVHD, and the grade III–IV of aGVHD, when assessed by Cox multivariate regression analysis. These clinical findings help us understand animal models (30, 44), suggesting that donor NK cells could play a regulatory role in GVHD by inhibiting allo-reactive T cell immune through their cytotoxic functions against activated allo-reactive T cells.

As NK cells may serve as potentially GVHD regulatory cells, studies have sought to determine the predictive value of NK cells in human GVHD. NK cell concentrations in allograft procedures are important factors influencing GVHD incidence (45–49). Tanaka et al. reported that a high dose of infused NK cells was correlated with a lower incidence of grade III–IV aGVHD, particularly in recipients receiving unrelated bone marrow transplantation (49). However, in our transplant settings, although higher NK doses in grafts showed correlations with a lower incidence of overall aGVHD by univariate analysis, higher NK cell doses in allografts were not identified as independent predictors of aGVHD using multivariate analysis. We speculated on several possible factors that may have contributed to this inconsistency. Firstly, there were large differences in infused NK cell doses in different transplantation schemes, varying from 10^6^ to 10^7^/kg, and NK content in PBSC harvests was usually higher than bone marrow collections (45). Secondly, different conditioning-regimens and GVHD prevention schemes may have exerted different effects on NK functions (50–52). Finally, and most importantly, the statistical significance of NK cell doses were weakened after NK cytotoxic function was incorporated into the multivariate model.

Zhao et al. observed that a higher dose of CD56^bright^ NK cells in allografts was associated with a higher incidence of grade II–IV aGVHD, while a higher CD56^dim^/CD56^bright^ ratio dose in NK cells was correlated with a lower incidence of grade III–IV aGVHD, after haplo-identical transplantation without *in vitro* T-cell depletion (48). When analyzing the relationship between NK cell subsets and aGVHD, we found no significant correlations between the CD56^dim^/CD56^bright^ ratio and aGVHD. Interestingly, we observed that a higher ratio of NKG2A^+^ NK in allografts was associated to a higher incidence of grade III–IV aGVHD. Equally, we showed that NKG2A was involved in the negative regulation of NK cell cytotoxicity against activated T cells *in vitro*, which was consistent with Nielsen et al. (36, 53). NKG2A, is an inhibitory receptor of NK cells which belongs to the C-type lectin superfamily, and is often overexpressed on the surface of reconstituted NK cells in the early stages after HSCT (54–56). Contrary to our results, Hu et al. reported that NKG2A^+^ subset cells were reduced in patients with aGVHD after allo-HSCT (54, 57). We speculated that the main reason for this inconsistency might be that Hu et al. studied the expression of NKG2A in reconstituted NK cells after transplantation, while we studied the expression of NKG2A on the surface of donor NK cells, while the phenotype and function of the NKG2A^+^ NK cells after allo-HSCT are different from those of healthy donors (55, 58).

Several mechanisms have been proposed to explain the complex crosstalk between NK cells and T cells during NK cell-mediated negative modulation of T cell immunity, including cytokine interactions, indirect effects by killing APCs, and the direct lysis of activated T cells (6, 28, 42, 59). This latter mechanism has been proposed as a direct mechanism used by NK cells (35, 40, 60), and several receptor-ligand pairs have been reported to manipulate NK cytotoxicity toward activated T cells, including NKG2D/NKG2D-L (25), DNAM-1/PVR (26), LFA/LFA-L (36), and NKP46/NKP46-L (29, 61). In accordance with previous reports (29, 36, 62), our results showed that NK cytolysis of allo-activated T cells depends on NKG2D, DNAM-1, and NKP46, as blocking of NKG2D, DNAM-1(CD226), or NKP46 led to significant reductions in degranulation of NK cells toward activated auto-T cells.

Several studies have demonstrated regulatory roles of NK cells in T cells responses in chronic viral infection (34), auto-immunity (63), transplantation (38, 64), and GVHD mouse models (30). Here, we specifically investigated NK-T cell crosstalk in a human GVHD setting. We have provided new insight into the role of NK cell “induced-self” recognition in aGVHD regulation. The triggering of NK cytotoxicity is tightly controlled by activating and inhibiting signals from NK cell receptors, the “missing-self” and “induced-self” recognition have been proposed to interpret the manner of NK activation (22, 65–67). The recognition of homologous HLA class I ligands by inhibitory KIR plays an important role in the education and self-tolerance of NK cells, which allows them to tolerate self-healthy cells with normal levels of HLA class I expression, but react to unhealthy cells with decreased HLA class I expression (68). When donor NK cells encounter autogenous allo-reactive T cells, the “missing-self” recognition model, which is triggered by KIR/KIR-ligand mismatch (20, 69), were prohibited as licensed NK cells expressing inhibitory KIR to combine with self HLA class I ligands on autogenous allo-reactive T cells (33, 70–72). It has been reported that activated T cells up-regulate ligands for NK cell activating receptors, and provide activating signal for autologous NK cells (28, 29, 60). When activating signals are strong enough to exceed the inhibitory signal from inhibitory KIR, the “induced-self” model of NK cell activation functions, and triggers cytotoxicity to eliminate redundant activated T cells, thus avoiding hyper T-cell activation and maintaining immune responses.

Donor allo-reactive T cells are an important factor leading to GVHD, and also a key compartment in exerting the graft-vs-leukemia (GVL) effect. Our concern is whether the negative regulatory effect of NK cells on allo-reactive T cells will affect GVL effect and increase disease relapse. In our study, we found that the cytotoxicity of NK cells on allo-reactive T cells did not affect the GVL effect. On the contrary, patients with higher NK degranulation activities toward allo-reactive T cells had a lower incidence of relapse, which was consistent with the results of previous studies that NK cells had the effect of separating GVHD from GVL (13, 73–75). However, the specific mechanism for donor NK cells separating GVL effect from GVHD is not clear. It is worth mentioning that NK cells themselves possess the powerful function of killing leukemia cells and prevent the relapse (76).

The present study had several limitations. First, the cohort of patients included in the study is heterogeneous as far as the donor source and ATG usage were concerned. Although we have conducted subgroup analysis, the limited number of cases may lead to the deviation of results, so we need to further validate the prognostic value of donor NK cell cytotoxicity toward allo-reactive T cells in a larger cohort of patients. Second, we did not find that donor NK cell cytotoxicity toward allo-reactive T cells was related to the severity of aGVHD, because there was no difference between the prognostic value of NK activity on the development of overall aGVHD and grade III–IV severe aGVHD, which may also be due to the limited number of cases and heterogeneous cohort. Third, other mechanisms for NK cells to regulate allo-reactive T cells, and the potential mechanism for NK cells to separate GVL from GVHD need to be explored in the future to provide further explanations for our findings.

In summary, donor NK cells inhibit and lyse allo-reactive T cells associated with aGVHD risk and severity, suggesting that NK cytotoxicity toward allo-activated T cells may play important roles in human aGVHD regulation. These findings may help us forecast aGVHD risks earlier by detecting donor NK cytotoxicity to allo-activated T cells, thus providing new targets for the prevention and treatment of aGVHD. However, it has been reported that NK cells reconstructed after allogeneic hematopoietic stem cell transplantation showed immature phenotypes and impaired functions (50, 58, 77, 78). Whether reconstructed donor NK can effectively regulate GVHD through cytotoxic function after allogeneic hematopoietic stem cell transplantation should be doubted, and more studies should be conducted to support this thesis.

## Data Availability Statement

The datasets generated for this study are available on request to the corresponding author.

## Ethics Statement

The studies involving human participants were reviewed and approved by The Clinical Ethics Review Committee at Ningbo First Hospital. Written informed consent to participate in this study was provided by the participants' legal guardian/next of kin. Written informed consent was obtained from the individual(s), and minor(s)' legal guardian/next of kin, for the publication of any potentially identifiable images or data included in this article.

## Author Contributions

LS, QM, HZ, and GO: conceptualization. LS, SY and GO: data curation. LS, QM, SY, and GO: formal analysis. LS, QM, and GO: funding acquisition. LS, QM, HF, YH, and GO: investigation. LS, SY, HF, YH, KX, YoS, YaZ, PZ, MZ, BL, ZX, MG, JW, YL, and YiZ: methodology. LS, QM, HZ, YW, and GO: project administration. LS, SY, and JW: software. All authors contributed to the article and approved the submitted version.

## Conflict of Interest

The authors declare that the research was conducted in the absence of any commercial or financial relationships that could be construed as a potential conflict of interest.

## Supplementary Material

Figure S1 Lineage-specific analysis of chimerism in patients following allogeneic stem cell transplantation. The peripheral blood samples of 38 patients were collected at 2, 4, 8, and 12 weeks after transplantation, then NK and T cells were enriched by immunomagnetic separation. The chimerism of NK and T cells was detested by short tandem repeats-Polymerase chain reaction (STR-PCR). **(A)** Chimerism dynamics of donor NK cells, **(B)** Chimerism dynamics of donor T cells.

Figure S2 Cytokine and granzyme B secretion of NK cells. After co-culture of NK cells with the unstimulated T cells or activated T cells for 4 h, the cytokine IFN-γ, TNF-α, IL-10, and TGF-β level secreted by NK cells was detected by flow cytometry. Representative gating strategy **(A)** and statistical histogram of four independent experiments **(B)** were shown (*n* = 4). The granzyme B were quantified by ELISA in supernatants after co-culture of NK cells with the unstimulated T cells or activated T cells for 4 h (*n* = 4) **(C)**.

Figure S3 Representative histograms for surface expression of ligands for NKG2D, DNAM-1, and NKG2A on activated and resting T cells.
