# Supplementary figures and images for: Cytotoxicity of Donor Natural Killer Cells to Allo-Reactive T Cells Are Related With Acute Graft-vs.-Host-Disease Following Allogeneic Stem Cell Transplantation
Source: Front Immunol. 2020 Jul 31;11:1534. doi: 10.3389/fimmu.2020.01534 (PMC7411138; doi:10.3389/fimmu.2020.01534)

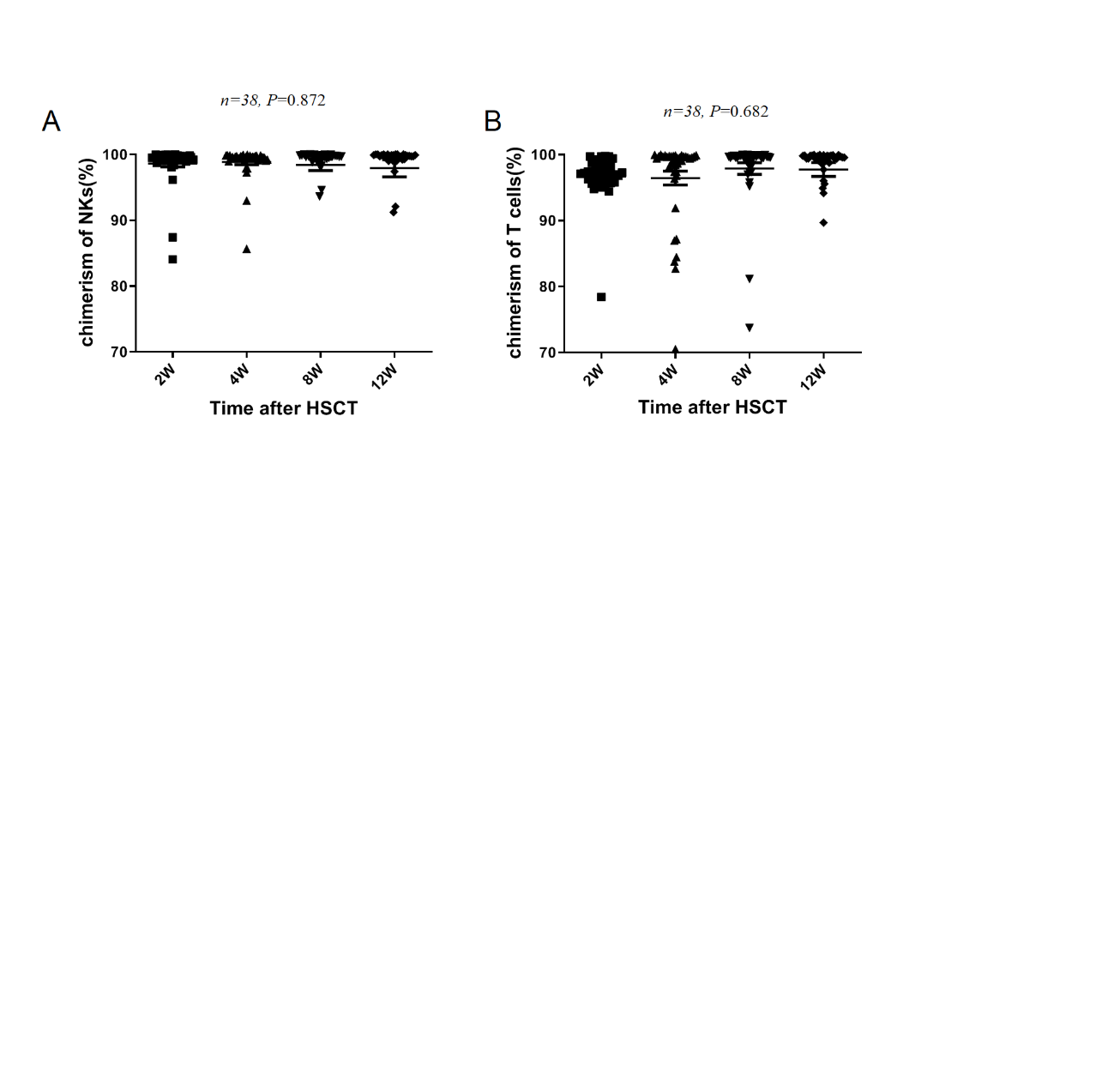

Supplement: Figure S1 — Lineage-specific analysis of chimerism in patients following allogeneic stem cell transplantation. The peripheral blood samples of 38 patients were collected at 2, 4, 8, and 12 weeks after transplantation, then NK and T cells were enriched by immunomagnetic separation. The chimerism of NK and T cells was detested by short tandem repeats-Polymerase chain reaction (STR-PCR). (A) Chimerism dynamics of donor NK cells, (B) Chimerism dynamics of donor T cells. [file Image_1.TIF]

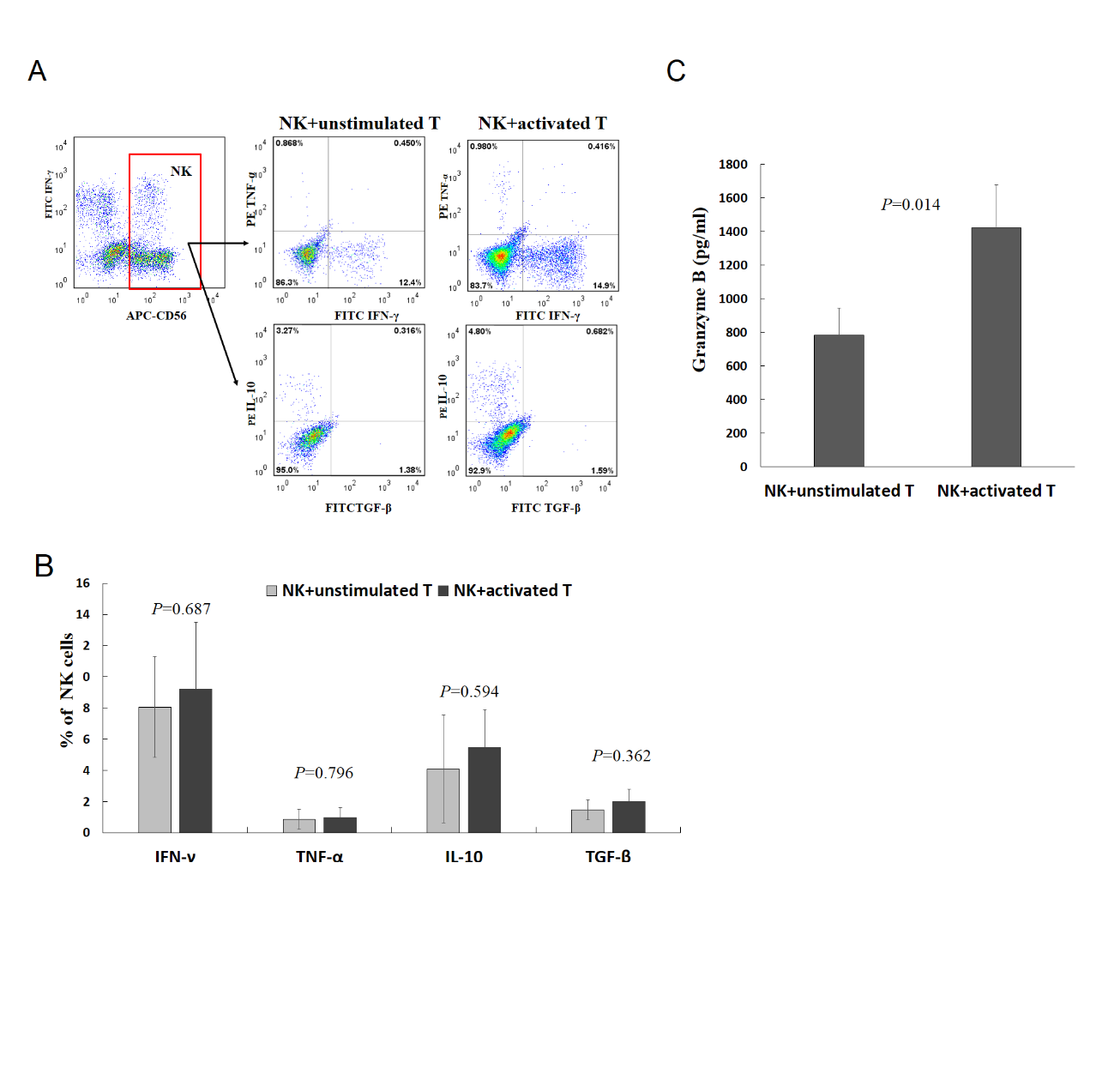

Supplement: Figure S2 — Cytokine and granzyme B secretion of NK cells. After co-culture of NK cells with the unstimulated T cells or activated T cells for 4 h, the cytokine IFN-γ, TNF-α, IL-10, and TGF-β level secreted by NK cells was detected by flow cytometry. Representative gating strategy (A) and statistical histogram of four independent experiments (B) were shown (n = 4). The granzyme B were quantified by ELISA in supernatants after co-culture of NK cells with the unstimulated T cells or activated T cells for 4 h (n = 4) (C). [file Image_2.TIF]

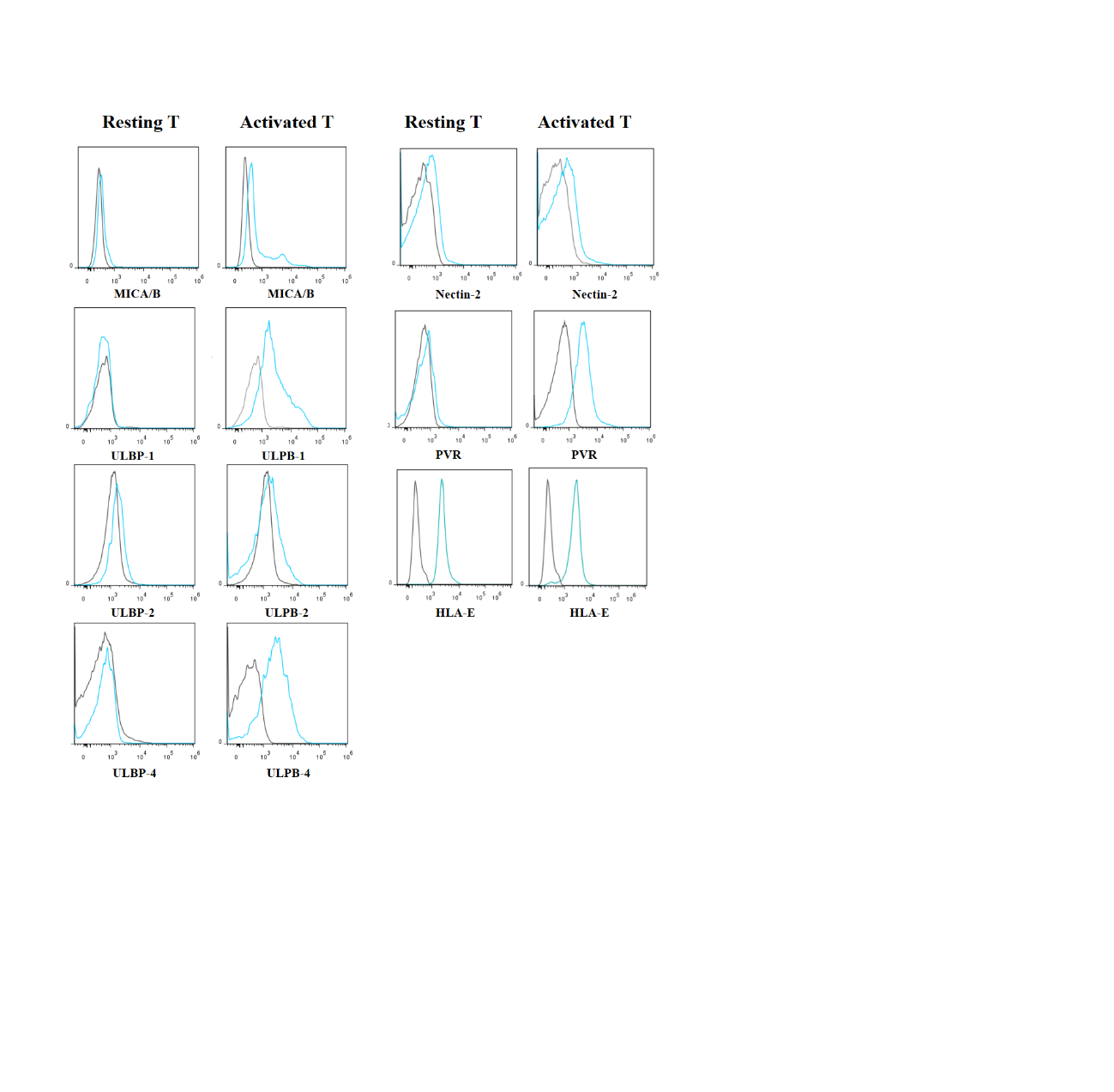

Supplement: Figure S3 — Representative histograms for surface expression of ligands for NKG2D, DNAM-1, and NKG2A on activated and resting T cells. [file Image_3.TIF]
